# Retraction: Curcumin alleviates osteoarthritis in mice by suppressing osteoclastogenesis in subchondral bone via inhibiting NF-κB/JNK signaling pathway

**DOI:** 10.1371/journal.pone.0340040

**Published:** 2026-01-08

**Authors:** The PLOS One Editors

After this article [1] was published, concerns were raised about Figs 3, 4, and 7. Specifically, the following figure panels appear similar:

- Fig 3D RANKL‑100 ng/ml CUR 10 μM day 3 panel of [1] and Fig 6A Sham+PBS panel of [2, retracted in 3].
- Fig 4C RANKL-100 ng/ml CUR-0 μM panel and the central region of the Fig 4C RANKL-100 ng/ml CUR-10 μM panel when enlarged.
- Fig 7A 8 weeks OA+CUR panel* of [1] and Fig 7B 8 weeks DMM+Met panel of [4, retracted in 5].
- Fig 7B 4 weeks OA+vehicle panel* of [1] and Fig 7C 4 weeks DMM + Met panel of [4], though it is noted that some features appear to differ between the differently stained tissue images.
- Fig 7E left panel* of [1] and Fig 7A 4 weeks sham-operated panel of [4]; upon editorial follow-up, the first author noted they consider this does not affect the experimental conclusion because both panels report data for the sham-operated group in each study.
- Fig 7E middle panel* of [1] and Fig 7A 8 weeks DMM + Met panel of [4], though it is noted that some features appear to differ between the two panels.
- Fig 7E right panel* of [1] and Fig 7A 4 weeks DMM + Met panel of [4].

Upon editorial follow-up, the authors did not provide underlying image data for the above-listed panels published in [1].

PLOS additionally noted concerns about adherence to PLOS policy on Animal Research. Specifically, the ethical approval document submitted with [1] appears to reference a study title that does not align with the study described in the manuscript. Additionally, the approval document is dated after the study period during which the animal research was conducted and the protocol period indicated in the ethics document is approximately one month, while the data presented in [1] span eight weeks. Upon editorial follow-up, the first author indicated that the study stemmed from a research project under a different title. We regret that the issues were not addressed prior to the article’s publication.

In light of the above concerns, which have not been resolved, the *PLOS One* Editors retract this article.

All authors agreed with the retraction and apologize for the issues with the published article.

The Fig 3D RANKL‑100 ng/ml CUR 10 μM day 3 panel reports material that is similar to that previously published in [2] and the above panels flagged with * report materials that are similar to those published in [4]. [2,4] were both published under a CC BY 4.0 license.
